# FURTHER EVALUATION OF A MODIFIED VERSION OF THE HOSPICE PHILOSOPHY SCALE: A DIFFERENTIAL ITEM FUNCTIONING ANALYSIS

**DOI:** 10.1093/geroni/igad104.3365

**Published:** 2023-12-21

**Authors:** Todd Becker, Sarah Clem, Paul Sacco, John Cagle, Joan Davitt, Nancy Kusmaul, Cindy Cain

**Affiliations:** Washington University in St. Louis, St. Louis, Missouri, United States; University of Maryland School of Social Work, Baltimore, Maryland, United States; University of Maryland, Baltimore, Baltimore, Maryland, United States; University of Maryland, Baltimore, Baltimore, Maryland, United States; University of Maryland, Baltimore, Baltimore, Maryland, United States; University of Maryland Baltimore County, Baltimore, Maryland, United States; University of Alabama at Birmingham, Birmingham, Alabama, United States

## Abstract

The hospice philosophy of care (HPOC) is a set of values applicable to all interdisciplinary group members that drives organizational and individual approaches to hospice care. As the only established measure of HPOC attitudes, we aimed to extend previous exploratory psychometric evaluation of the eight-item Hospice Philosophy Scale (HPS-8). An interdisciplinary convenience sample of 481 hospice clinicians participated in this cross-sectional study. Participants were recruited through hospice and palliative care membership associations representing the core members of the Medicare Hospice Benefit-designated hospice interdisciplinary group (physicians, nurses, social workers, chaplains). After examining uniformity through confirmatory factor analysis, we tested HPS-8 items for differential item functioning (DIF) by professional discipline using multiple indicators, multiple causes models. We compared models via nested chi-square difference tests. Our final model assessed uniform DIF for the HPS-8 factor, controlling for DIF-indicated items. We also assessed internal consistency reliability. After correlating errors for two similarly worded items, global fit indices met prevailing thresholds, χ²(19)=55.18, p<.001 (RMSEA=.06, SRMR=.03, CFI=.98, TLI=.97). The uniform DIF model indicated two items with DIF. Chaplains endorsed the need for mental and spiritual preparation for death more than physicians (β=0.21, p<.001). Nurses endorsed involving patients and families in deciding treatments and services more than physicians (β=0.21, p=.006). Despite statistically significant DIF on these items, statistically significant differences among professions were not observed at the factor level (p>.50 for all). Composite reliability estimates satisfied internal consistency thresholds (CR=.76). Results support the HPS-8’s use as a valid and reliable measure for assessing HPOC in hospice clinicians.

